# An Emerging Lineage of Uropathogenic Extended Spectrum β-Lactamase Escherichia coli ST127

**DOI:** 10.1128/spectrum.02511-22

**Published:** 2022-11-23

**Authors:** Rory Cave, Mary M. Ter-Stepanyan, Nune Kotsinyan, Hermine V. Mkrtchyan

**Affiliations:** a School of Biomedical Sciences, University of West London, London, United Kingdom; b Yerevan State Medical University after Mkhitar Heratsi, Faculty of Public Health, Department of Epidemiology, Yerevan, Republic of Armenia; c Research Center of Maternal and Child Health Protection, Yerevan, Armenia; d National Centre for Disease Control and Prevention, Yerevan, Armenia; University of Texas Southwestern Medical Center

**Keywords:** ESBL, extraintestinal pathogenic *E. coli* (ExPEC), ST127, UTI infections, uropathogenic *Escherichia coli* (UPEC), WGS

## Abstract

Uropathogenic Escherichia coli (UPEC) is one of the most common causes of urinary tract infections. Here, we report for the first time the whole-genome sequencing (WGS) and analysis of four extended-spectrum β-lactamase (ESBL), UPEC sequence type (ST) 127 isolates that were recovered from patients in five hospitals in Armenia from January to August of 2019. A phylogenetic comparison revealed that our isolates were closely related to each other by their core and accessory genomes, despite having been isolated from different regions and hospitals in Armenia. We identified unique genes in our isolates and in a closely related isolate recovered in France. The unique genes (hemolysin E virulence gene, lactate utilization operon *lutABC*, and endonuclease restriction modification operon *hsdMSR*) were identified in three separate genomic regions that were adjacent to prophage genes, including one region containing the TonB-dependent iron siderophore receptor gene *ireA,* which was only found in 5 other ST127 isolates from the European Nucleotide Archive (ENA). We further identified that these isolates possessed unique virulence and metabolic genes and harbored antibiotic resistance genes, including the ESBL genes *bla*_CTX-M-3_ (*n* = 3), *bla*_CTX-M-236_ (*n* = 1), and *bla*_TEM-1_ (*n* = 1), in addition to a quinolone resistance protein gene *qnrD1* (*n* = 1), which was absent in the ST127 isolates obtained from the ENA. Moreover, a plasmid replicon gene IncI2 (*n* = 1) was unique to ARM88 of the Armenian isolates. Our findings demonstrate that at the time of this study, E. coli ST127 was a cause of urinary tract infections in patients in different regions of Armenia, with a possibility of cross-country transmission between Armenia and France.

**IMPORTANCE** Whole-genome sequencing studies of pathogens causing infectious diseases are seriously lacking in Armenia, hampering global efforts to track, trace and contain infectious disease outbreaks. In this study, we report for the first-time the whole-genome sequencing and analysis of ESBL UPEC ST127 isolates recovered from hospitalized patients in Armenia and compare them with other E. coli ST127 retrieved from the ENA. We found close genetic similarities of the Armenian isolates, indicating that E. coli ST127 was potentially a dominant lineage causing urinary tract infections in Armenia. Furthermore, we identified unique genes that were horizontally acquired in the clusters of Armenian and French isolates that were absent in other ST127 isolates obtained from the ENA. Our findings highlight a possible cross-country transmission between Armenia and France and the idea that the implementation of WGS surveillance could contribute to global efforts in tackling antibiotic resistance, as bacteria carrying antimicrobial resistance (AMR) genes do not recognize borders.

## INTRODUCTION

Escherichia coli is one of the most common causes of urinary tract infections (UTI), and it is estimated to affect about 150 million people, globally (1). New, emerging lineages present a major challenge for health care settings due to their increased resistance to multiple antibiotics (2), which contributes to a large number of hospitalizations and associated costs. The uropathogenic E. coli (UPEC) pathotype is the primary cause of urinary tract infections and is a part of the broader pathotype designated extraintestinal pathogenic E. coli (ExPEC), which is known to cause infections in the bloodstream and in other nonintestinal sites (3). Further members of the ExPEC pathotype include neonatal meningitis E. coli (NMEC), sepsis-associated E. coli (SEPEC), and avian pathogenic E. coli (APEC) (4). One of the new emerging ExPEC pandemic genotypes of interest is sequence type (ST) 127, which is known for its high virulence and low antibiotic resistance potential, compared to other pandemic ExPEC sequence types (5–7). The acquisition of many virulence and antibiotic resistance determents by UPEC isolates occurs through the horizontal transfer of plasmids and prophages that can integrate into the bacterial chromosome (8–10). These acquired genes are only maintained long-term if they demonstrate positive evolutional adaption with little fitness cost under nonselective conditions (11). An example of this is the plasmid-borne CTX-M-type genes that encode extended-spectrum β-lactamases (ESBL), which give the bacteria resistance toward extended-spectrum cephalosporins and monobactams (12). Over the past decade, the prevalence of ESBL-producing E. coli has increased globally (13–15). However, ESBL-producing UPEC ST127 isolates are rarely reported in the literature (5, 16, 17).

Our understanding of UPEC infections has greatly improved due to the abilities of whole-genome sequencing (WGS) analyses to trace the sources and transmission routes of infections as well as to identify genes involved in virulence and antibiotic resistance for the implementation of improved infection control practices (6, 18–20). However, studies reporting WGS are limited to some regions of the world (i.e., the United Kingdom, the European Union, and North America), whereas studies from low-middle-income countries are fragmented (21, 22). There have been limited studies utilizing WGS in Armenia, an upper-middle-income country (23) (according to the World Bank in 2022), with none reported for E. coli. Previously, we reported the occurrence of diverse MRSA genotypes (24) and provided insights into the genomic background and phylogenetic origins of MRSA isolates in Armenia (25). In this study, we report for the first time the genetic features of ESBL UPEC ST127 isolates recovered from hospitalized patients in Armenia using WGS analysis, compare them with 168 other E. coli ST127 recovered from multiple sources (available at the European Nucleotide Archive [ENA]), and identify the mode of horizontal transfer of the unique genes found only in the Armenian isolates.

## RESULTS

### Isolates and antibiotic susceptibility testing.

4 out of the 12 sequenced E. coli isolates (Table S3) belonged to ST127 (Table 1) and were resistant to 7 (*n* = 1), 6 (*n* = 1), and 5 (*n* = 2) of the antibiotics tested. All four isolates were resistant to the cephalosporin antibiotics cefepime and ceftazidime as well as the aminopenicillins antibiotic ampicillin. In addition, 3 isolates (ARM64, ARM75, and ARM88) were resistant to the β-lactam antibiotic amoxicillin-clavulanic acid, and 2 isolates (ARM64 and ARM66) had intermediate resistance to the β-lactam antibiotic piperacillin-tazobactam. One isolate (ARM88) was resistant to the aminoglycoside antibiotic amikacin and the fluoroquinolone antibiotics norfloxacin and levofloxacin (ARM75). One isolate had an intermediate resistance to the carbapenem antibiotic imipenem (ARM66). All four isolates were sensitive to the β-lactam antibiotic meropenem and were also sensitive to chloramphenicol.

**TABLE 1:** Antibiotic susceptibility profiles of E. coli ST127 isolates recovered from urine specimen^*a*^

ST	ID	AMP	TZP	AMC	CAZ	CPM	NOR	LVX	AMK	IPM	MEM	CHL
127	ARM64	R	I	R	R	R	S	S	S	S	S	S
127	ARM66	R	I	S	R	R	S	S	S	I	S	S
127	ARM75	R	S	R	R	R	R	R	S	S	S	S
127	ARM88	R	S	R	R	R	S	S	R	S	S	S

*^a^*AMP, ampicillin; TZP, piperacillin-tazobactam; AMC, amoxicillin-clavulanic acid; CAZ, ceftazidime; CPM, cefepime; NOR, norfloxacin; LVX, levofloxacin; AMK, amikacin; IMP, imipenem; MEM, meropenem; CHL, clindamycin.

### Phylogenetic analysis of E. coli ST127.

**FIG 1:**
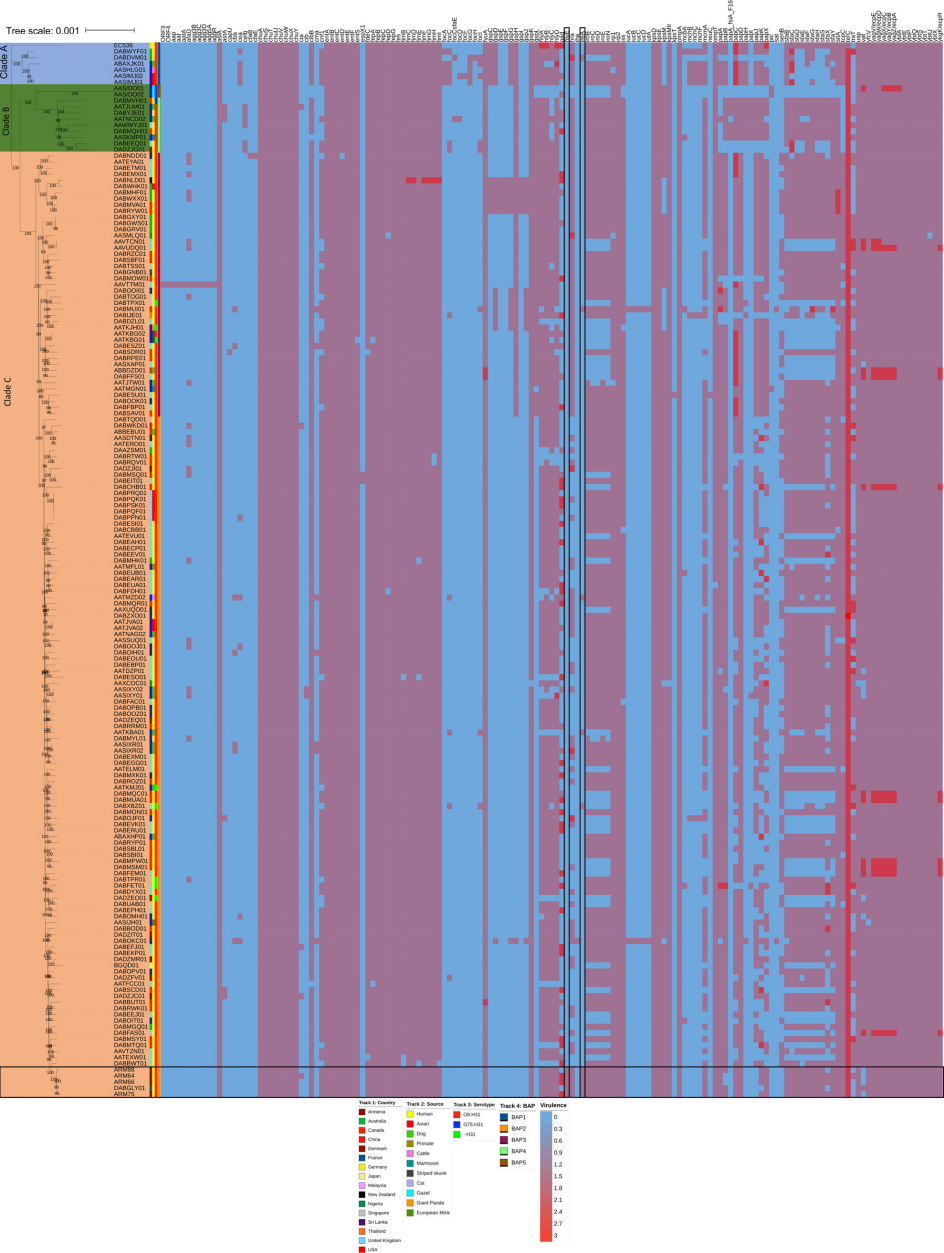
ST127 core maximum likelihood phylogenetic tree and virulence gene heat map. (A) Phylogenetic tree and virulence gene heat map. (B) Correlation matrix of virulent genes and their associations to different sources and clusters. The red circle in the phylogenetic tree represents the Armenian isolates.

The short reads of four of the E. coli ST127 in this study were aligned against 168 E. coli ST127 genomes that were obtained from the ENA archive and were previously recovered from 15 countries and 11 different animal sources and belonged to 3 different serotypes (O6:H31 [*n* = 161], O75:H31 [*n* = 3], and -:H321 [*n* = 8]) (Table 2). A maximum-likelihood phylogenetic tree of the core genome revealed that ST127 could be split into 3 main clades, with no particular clade having isolates that were only found from one particular country or source (Fig. 1A). All of the O75:H31 serotype isolates clustered together in clade B. Further detailed analyses of the clusters (using heirBAPS) revealed that the ST127 isolates could be further grouped into 5 distinct clusters. The E. coli ST127 isolates recovered in our study belonged to clade C BAP2 and were phylogenetically closely related to each other, with a maximum of 33 SNP differences between the isolates (Table S4). Moreover, all of the Armenian ST127 isolates were phylogenetically closely related to a French isolate (DABGLY01) that was recovered from a patient (urine sample) in 2015.

**TABLE 2:** Isolation source, country, and BAP cluster of E. coli ST127 used in the phylogenetic analysis

Country	Source	BAP
Sri Lanka (*n* = 1)	Human (*n* = 137)	BAPI (*n* = 7)
Armenia (*n* = 4)	Cattle (*n* = 1)	BAP2 (*n* = 111)
USA (*n* = 44)	Gazelle (*n* = 0)	BAP3 (*n* = 43)
Nigeria (*n* = 2)	European Mink (*n* = 1)	BAP4 (*n* = 9)
United Kingdom (*n* = 55)	Avian (*n* = 10)	BAP5 (*n* = 2)
Canada (*n* = 34)	Dog (*n* = 14)	
Japan (*n* = 1)	Marmoset (*n* = 2)	
Australia (*n* = 11)	Striped skunk (*n* = 2)	
France (*n* = 2)	Giant panda (*n* = 1)	
Thailand (*n* = 1)	Primate (*n* = 1)	
New Zealand (*n* = 1)	Cat (*n* = 1)	
Singapore (*n* = 4)		
Malaysia (*n* = 2)		
China (*n* = 1)		
Denmark (*n* = 8)		
Germany (*n* = 1)		

**FIG 2:**
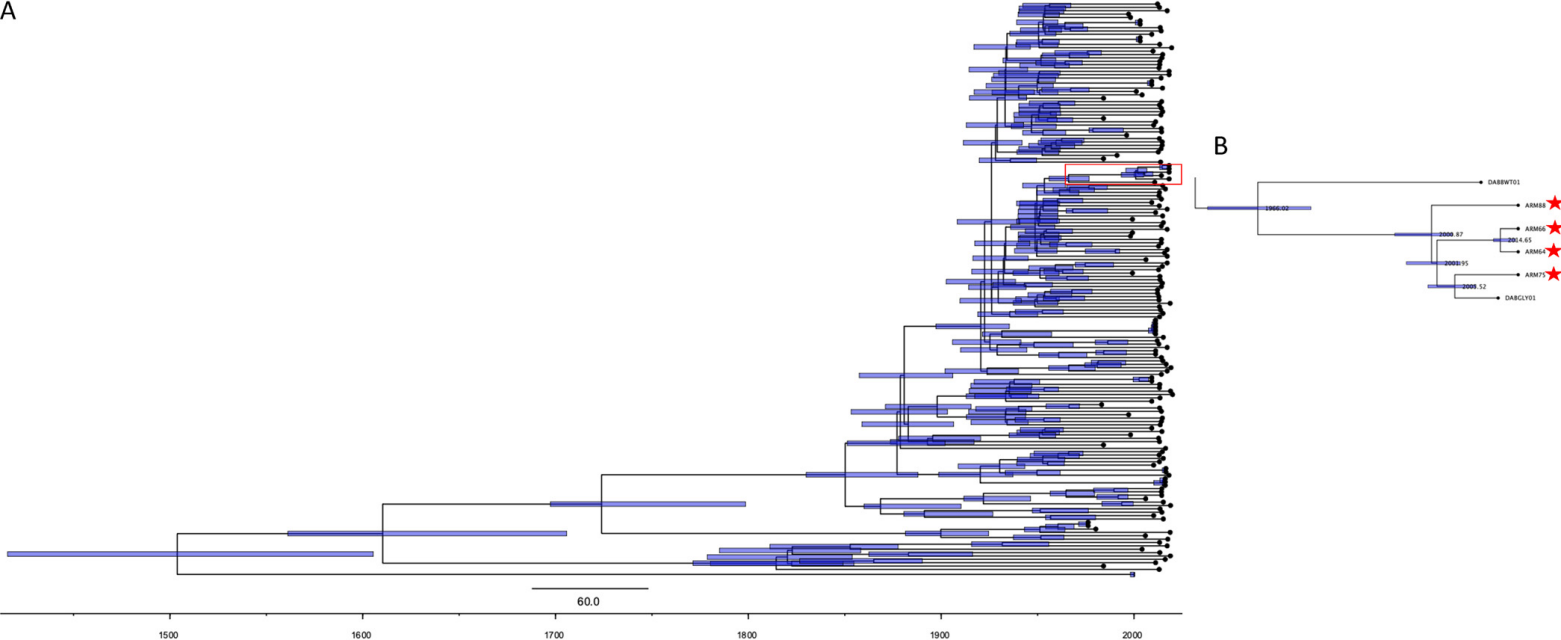
MCC time-calibrated phylogenetic tree of ST127 E. coli. The blue bars on the nodes represent the date of divergence 95% confidence intervals. (A) Full dated MCC tree. (B) Partial MCC time-calibrated phylogenetic tree, showing the most recent divergence.

A maximum clade credibility (MCC) time-calibrated phylogeny tree of 171 E. coli ST127 isolates (excluding the EC536 isolate due to the lack of its date of isolation) was constructed using BEAST to determine the inferred date of divergence of the Armenian isolates and the French isolate (DABGLY01) obtained from the ENA archive to other ST127 isolates (Fig. 2). The ST127 had a rate estimate of 9.18 × 10^−5^ substitutions per site per year and an inferred tree root date of 1503 (date confidence interval [CI]: 1415 to 1605). The inferred divergence date of these isolates to their closest phylogenetically related isolate (the UK isolate DABBWT01 recovered from blood) was 1966 (date CI: 1956 to 1977). The most recent divergence date that was shared by both the Armenian isolates and the French isolate was 2000 (date CI: 1995 to 2006), and the most recent divergence date between ARM75 and the closely phylogenetically related French isolate DABGLY01 was 2005 (date CI: 2000 to 2009).

### Comparison of E. coli ST127 virulence genes.

Overall, we identified 153 virulence genes in the E. coli ST127 isolates that we analyzed, of which 41 were ubiquities (Fig. 1B). The Armenian isolates harbored 96 (ARM64), 95 (ARM75), 94 (ARM66), and 92 (ARM88) virulence genes, and these numbers are less than the average number (average: 100 genes; range: 63 to 109 genes) of virulence genes, compared to the other ST127 isolates that were analyzed.

All four of the isolates in this study and a phylogenetically closely related French isolate DABGLY01 (<25 SNP difference between the Armenian isolates and the French isolate within the core genomes) harbored the hemolysin E gene (*hylE*), which was not present in any of the other ST127 isolates. In addition, they also carried the TonB-dependent iron siderophore receptor gene *ireA*, which was also found in five other ST127 isolates recovered from a dog (AATKBA01), a cat (AATMZD02), and human urine and fecal swabs (DABDVM01, DABMUI01, DABXBZ01). However, these isolates did not show any phylogenetic or geographical relationship to each other.

### Unique genes and origin of horizontal gene transfer.

**FIG 3:**
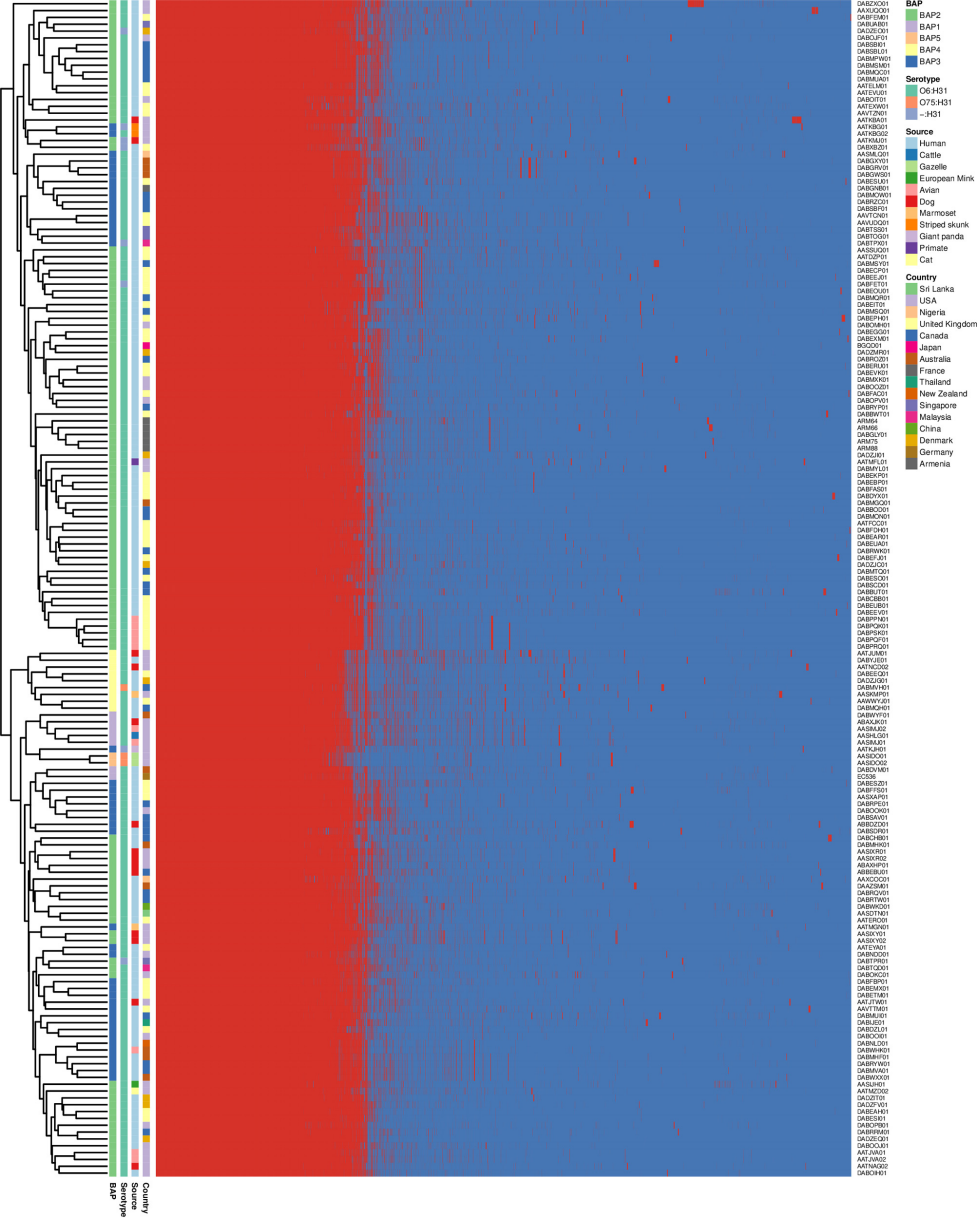
Hierarchy cluster heat map of the accessory genome of ST127 E. coli isolates.

To further determine whether any of the genes were unique to the Armenian isolates or to the French isolate obtained from the ENA archive, we conducted a pangenome analysis of all of the E. coli ST127 isolates. The pangenome of the E. coli ST127 isolates consisted of 14,720 genes, of which 3,529 belonged to the core genome and 11,191 belonged to the accessory genome. The accessory genome hierarchy clustering heat map (Fig. 3) showed that the Armenian isolates shared many accessory genes with DABGLY01.

Using Scoary, we identified 16 genes that were unique to all of the E. coli ST127 Armenian isolates and also to DABGLY01, which was recovered in France. 11 out of the 16 genes identified had a known function (Table 3), including the hemolysin E toxin (*hylE*), lactate utilization protein operon (*lutABC*), peptide ABC transporter substrate-binding protein, transposase *insD* for insertion element IS2, Inovirus Gp2 family protein, and endonuclease restriction system operon (*hsdMSR*). We identified that 12 of these genes were located within the same locus, next to a prophage integrase *intA* gene, including the *lutABC* operon gene and the *oppA* gene. In addition, two genes that were located near the *ireA* gene and were unique to the Armenian and DABGLY01 isolates are *InsD* and the Inovirus Gp2 family protein gene. Moreover, we found one additional uncharacterized gene that was unique to the Armenian isolates adjacent to the *hlyE* virulent gene.

**TABLE 3:** Unique genes (with known functions) detected in ARM64, ARM66, ARM75, ARM88, and DABGLY01

Gene	Function
No name	DNA binding protein
*hsdM*	Site-specific DNA-methyltransferase (adenine-specific)
*hsdS*	Restriction endonuclease subunit S
*lutC*	Lactate utilization protein C
*lutB*	LutB/LldF family l-lactate oxidation iron-sulphur protein
*lutA*	(Fe-S)-binding protein
No name	peptide ABC transporter substrate-binding protein
*hlyE*	Hemolysin E toxin
*hsdR*	Type I restriction enzyme R Protein
*InsD*	Transposase *InsD* for insertion element IS2A/D/F/H/I/K
Gp2	Inovirus Gp2 family protein

**FIG 4:**
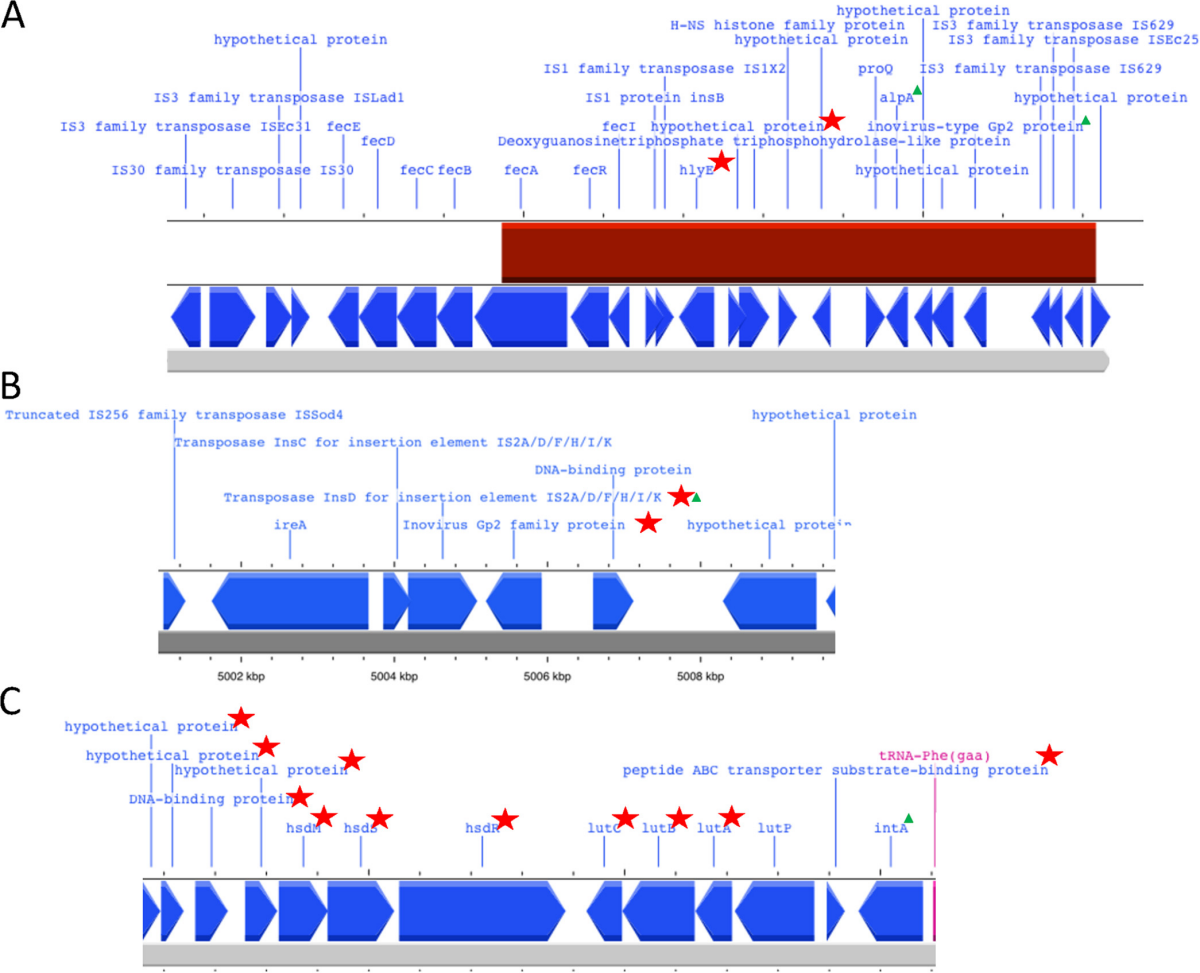
Genome loci of unique genes found within the Armenian isolates ARM64, ARM66, ARM75, ARM88, and the French isolate DABGLY01. (A) *hlyE* loci. (B) *ireA* loci. (C) *lutABC/hsdMSR* operon loci. Red star, unique genes to these isolates; green triangle, prophage genes; red block, prophage region identified by Prophage Hunter.

To determine the origin of the horizontal gene transfer of these unique genes found in the Armenian isolates, we looked for insertion via transposable elements, such as integrated and conjugated plasmids within the chromosome and phage insertions. Using the MOB-suite software package to reconstruct plasmids from WGS data, we determined that all of these genes were chromosomal and were not plasmid-borne. Further, using ICEfinder, we determined that these unique genes did not belong to integrative and conjugative elements (ICEs). Using Prophage hunter, we identified that the *hlyE* gene found in the Armenian isolates was located within the active prophage region (Fig. 4A), with the closest match being to Stx2-converting phage Stx2a_F349 (96% homology and 9% coverage of the prophage gene region). The *ireA* gene region (Fig. 4B) and the *lutABCP/hsdMSR* operons gene region (Fig. 4C) were not predicted to be prophage insertion regions by Prophage Hunter. However, based on genes found within these loci (the unique Inovirus Gp2 family protein gene, which was found both in the isolates of this study and in DABGLY01 [recovered in France] and was adjacent to the *ireA* gene and to the *lutABC* and *hsdMSR* operons that are adjacent to the prophage integrase gene *intA* and near tRNA-phe), we hypothesize that a possible horizontal transfer of genes could have occurred in these regions via prophages.

**FIG 5:**
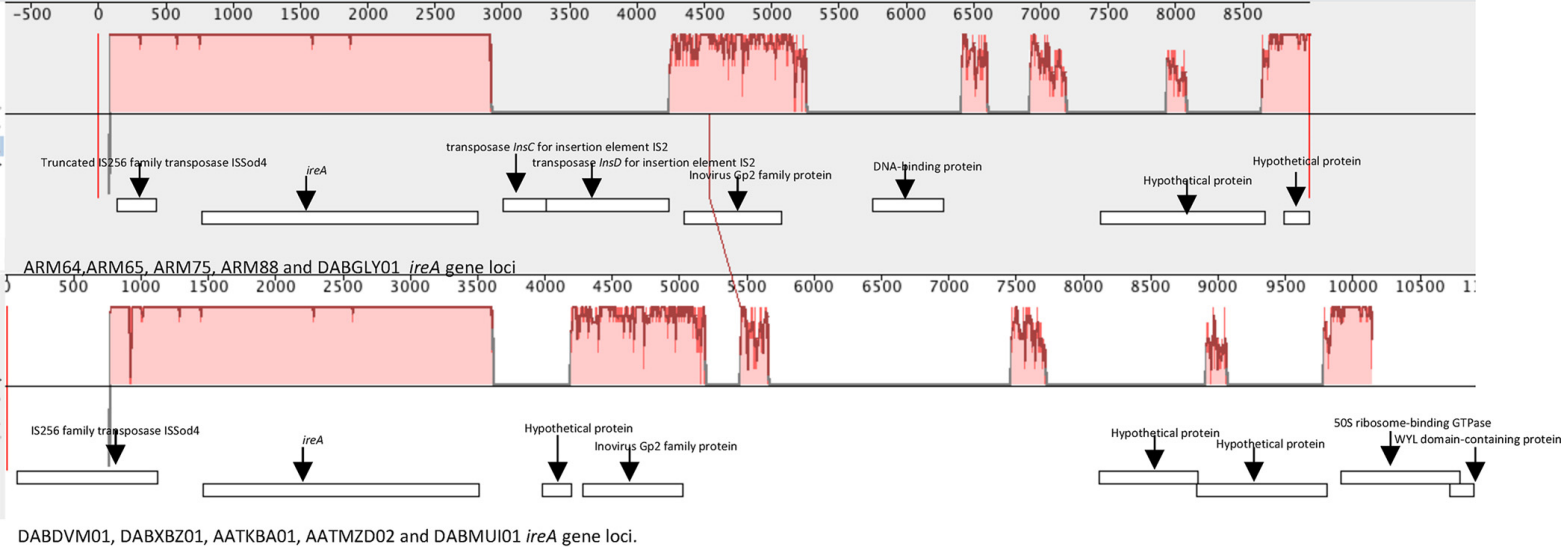
Mauve output of genome loci comparison of the *ireA* gene. The red block indicates genome similarities between the two loci.

The *ireA* virulent gene was present in other E. coli ST127 isolates, but the adjacent *incD* and the Inovirus Gp2 protein, which were unique to the Armenian and DABGLY01 isolates, were absent. To investigate further, we conducted a comparative analysis of the *ireA* gene loci of all of the E. coli ST127 isolates. The main difference in the *ireA* gene loci was that all of the isolates recovered in our study and DABGLY01 harbored two additional insertion sequence transposases instead of a hypothetical protein gene. In addition, the Inovirus Gp2 protein family gene found in the *ireA* locus shared 87.24% protein similarity (Fig. 5).

To determine whether the unique genes and genomic regions detected in the Armenian (ARM64, ARM66, ARM75, and ARM88) and French (DABGLY01) isolates were also present in other E. coli isolates from different sequence types, we conducted a sequence similarity search against the entire ENA archive (Tables S5, S6, and S7). We found that each unique genomic region had 100% homology with E. coli isolates belonging to other sequence types. In addition, the *ireA* (Table S5) and *hlyE* (Table S6) gene regions had 100% homology with those found in E. coli ST761 and ST93 isolates recovered from pig feces in Canada and chicken meat in South Korea, respectively. For the *lutABC*/*hsdMS* genomic region (Table S7), we found multiple E. coli isolates from the ENA archive that had 100% homology with the Armenian ST127 isolates that were sequence types ST1485, ST40, ST117, ST1266, ST95, and ST117, isolated from China, Japan, the United States of America, France, Switzerland, and the United Kingdom and from mammalian, avian, human, and wastewater sources. However, we did not find isolates with ≥99.99% homology that contained all three genomic regions; instead, we detected multiple isolates that harbored the *ireA* and *hlyA* genomic region and the *ireA* and *lutABC*/*hsdMSR* genomic region. However, all three genomic regions were identified in various isolates belonging to the ST117 sequence type.

### Antibiotic resistance genotype.

**FIG 6:**
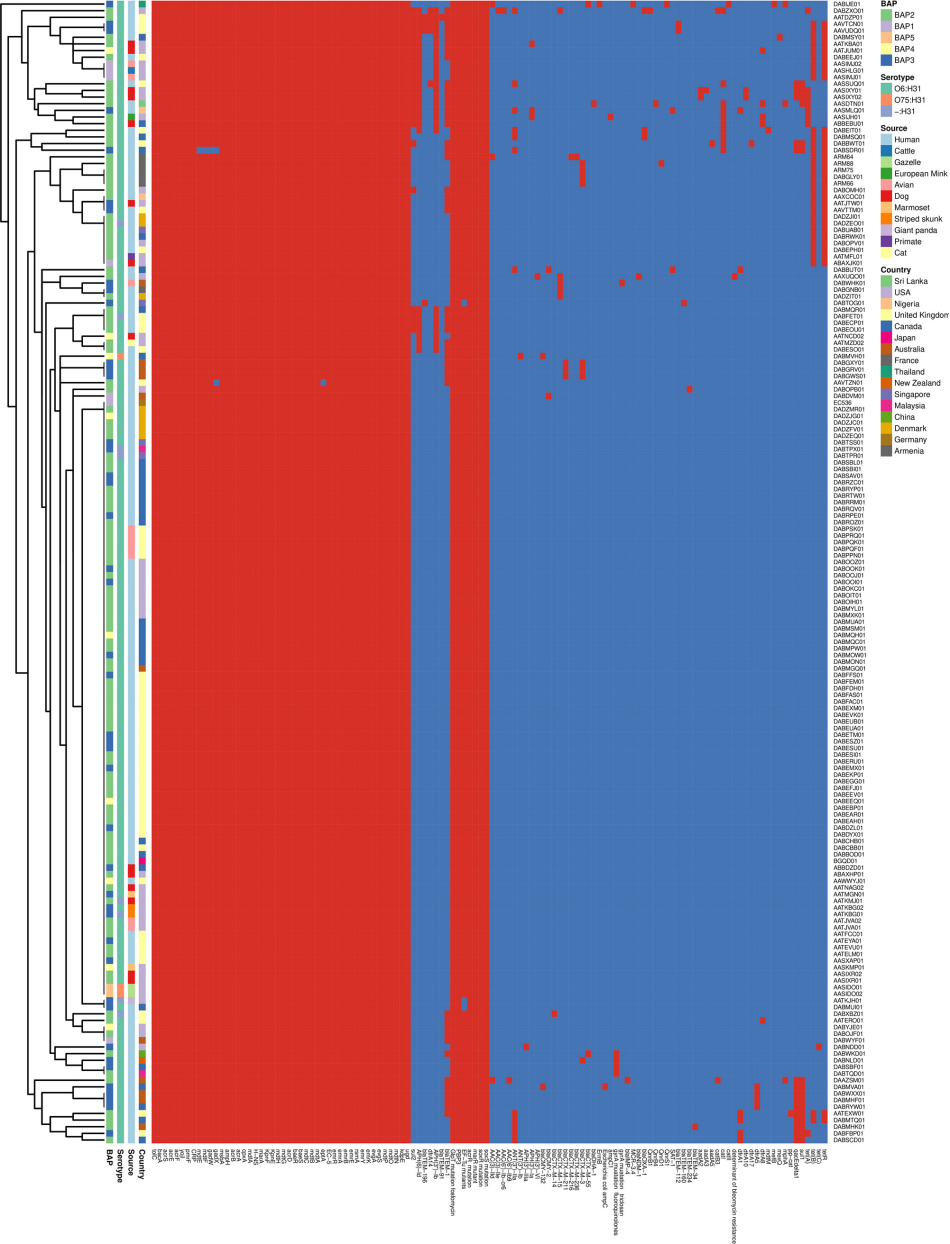
Hierarchy cluster heat map of ST127 E. coli antibiotic-resistant gene/mutation.

The antimicrobial resistance genes and mutations of all of the Armenian E. coli ST127 isolates were compared to 168 WGS data sets of E. coli ST127 isolates obtained from the ENA archive. In total, we identified 120 different antimicrobial genes/mutations within the resistome of these isolates (Fig. 6). 47 of these genes/mutations were present in all 172 of the E. coli ST127 isolates that we analyzed. These core antibiotic resistance genes/mutations are known to induce resistance to antibiotics, including those belonging to the aminocoumarin, aminoglycoside, carbapenem, cephalosporin, cephamycin, diaminopyrimidine, fluoroquinolone, fosfomycin, glycylcycline, macrolide, monobactam, nitroimidazole, nucleoside, penam, peptide, phenicol, rifamycin, and tetracycline classes.

All four of the isolates that were recovered in this study possessed various degrees of antibiotic resistance genes/mutations, including 1 isolate (ARM64) possessing 59 antibiotic resistance genes/mutations, 1 isolate (ARM88) possessing 57 antibiotic resistance genes/mutations, and 2 isolates (ARM66 and ARM75) possessing 56 antibiotic resistance genes mutations. These numbers are greater than the average number of antibiotic resistance genes, compared to the E. coli ST127 isolates obtained from the ENA archive (average: 55 genes; range: 52 to 67 genes) that have been reported previously. In addition, a plasmid-mediated quinolone resistance protein gene *qnrD1* was unique to the ARM88 isolate recovered in this study.

60 out of the 172 (34.88%) ST127 isolates, and all 4 of the Armenian isolates that were recovered in this study, possessed genes that encode ESBL, including but not limited to: CTX (*n* = 17), TEM (*n* = 48), and OXA-1 (*n* = 3). The most common CTX gene was *bla*_CTX-M-3_ (*n* = 16), which was also found in 3 of the Armenian isolates (ARM66, ARM75, and ARM88), *bla*_CTX-M-15_ (*n* = 15), *bla*_CTX-M-211_ (*n* = 6), *bla*_CTX-M-55_ (*n* = 4), *bla*_CTX-M-216_ and *bla*_CTX-M-236_ (*n* = 2), which was found in one of the Armenian isolates (ARM64), and *bla*_CTX-M-14_ (*n* = 2). The most common TEM gene was *bla*_TEM-1_ (*n* = 45), which was also found in ARM64, *bla*_TEM-196_ (*n* = 4), *bla*_TEM-112_ (*n* = 2), *bla*_TEM-160_ (*n* = 1), *bla*_TEM-234_ (*n* = 1), *bla*_TEM-34_ (*n* = 1), and *bla*_TEM-91_ (*n* = 1). In addition, the carbapenem-resistance genes *bla*_NMD-1_ (*n* = 1) and *bla*_IPM-4_ (*n* = 1) were detected in one of each of the E. coli ST127 isolates. However, they were detected only in those obtained from the ENA archive. We also found the antibiotic resistance genes/mutations, which were found in nearly all of the E. coli ST127 isolates from the ENA archive and in the all of the Armenian E. coli ST127 isolates, including the multidrug efflux *mdtE*, *mdtF*, the *gadW* and *gadX*, the pmr phosphoethanolamine transferase gene *eptA* (found in 171 of the 172 isolates, except for *gadX*, which was found in 170 of the 172 isolates), the elfamycin antibiotic resistance EF-Tu mutation (169 of the 172 E. coli ST127 isolates) and the tetracycline efflux pump genes *tet(B)* and *tetR* (32 of the 172 E. coli ST127 isolates). In addition, the aminoglycoside acetyltransferase gene *AAC(3)-IId* was identified in ARM64 (3 of the 172 E. coli ST127 isolates).

### Plasmid typing.

**FIG 7:**
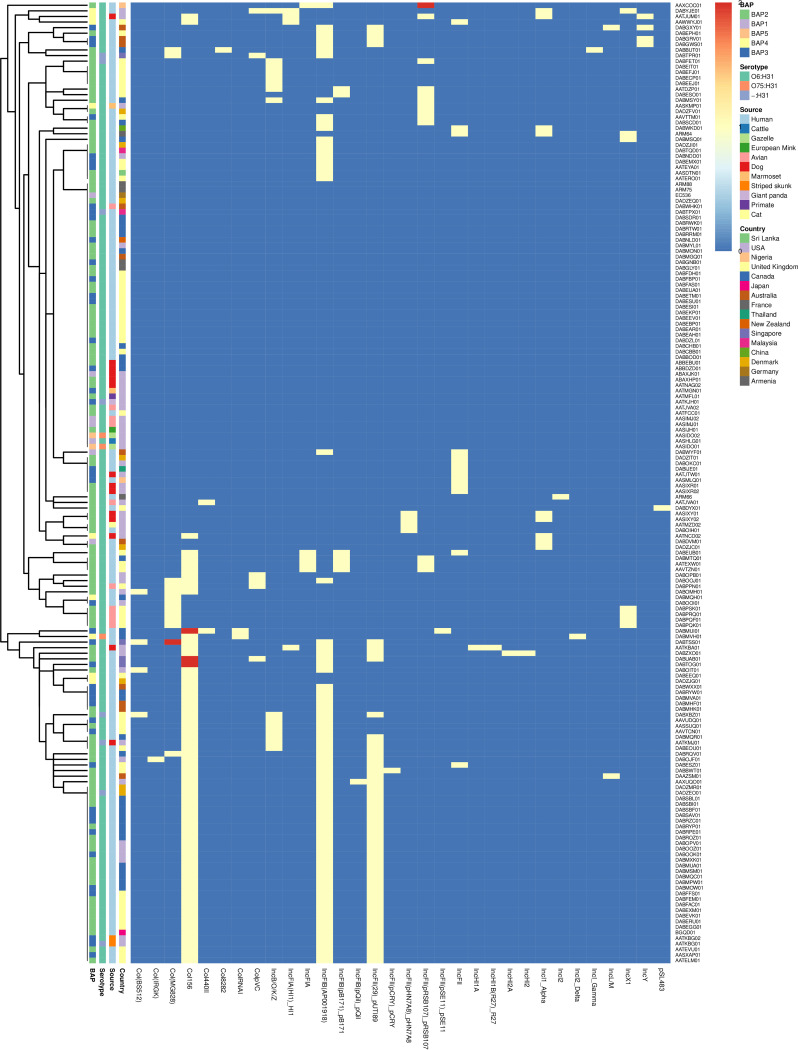
Hierarchy cluster heat map of ST127 E. coli plasmid replicon.

A plasmid replicon was identified in 72% (124/172) of the E. coli ST127 isolates, including in two Armenian isolates (ARM64 and ARM66). 36 different plasmid replicons were identified, of which IncFIB (AP001918) 1 was the most common (*n* = 74), followed by Col156_1 (*n* = 71) and IncFII (26) 1_pUTI89 (*n* = 48) (Fig. 7). The greatest number of plasmid replicons identified was 6 (*n* = 2), followed by 5 (*n* = 6), 4 (*n* = 17), 3 (*n* = 48), 2 (*n* = 20), and 1 (*n* = 31). The ARM64 isolates harbored 3 plasmid replicons, including IncFII_1 (found in 8.14% of the ST127 isolates), IncI1_1_Alpha (found in 5.23% of the ST127 isolates), and IncX1 (found in 4.07% of the ST127 isolates). Moreover, ARM66 harbored one plasmid replicon, IncI2_1, that was unique to it. Overall, we found that isolates, based on the hierarchy cluster heat map of their plasmid replicons, do not group with their BAP cluster groups. We did not detect any known plasmid replicon for the ARM75 and ARM88 isolates, despite MOB-suite identifying part of the genome sequence as plasmid-associated.

## DISCUSSION

Studies investigating the potential of the DNA sequencing of pathogens that cause infectious diseases are seriously lacking in Armenia, hampering global efforts to track, trace, and contain infectious disease outbreaks. In this study, we report for the first-time a whole-genome analysis of ESBL UPEC ST127 isolates recovered from hospitalized patients in Armenia, compare them with other E. coli ST127 retrieved from the ENA (recovered from multiple sources), and identify the mode of horizontal transfer of the unique genes found only in the Armenian isolates. Interestingly, all of the Armenian E. coli ST127 isolates were identified as UPEC ESBL and belonged to the same genetic lineage. Despite these isolates being recovered from patients in five different hospitals, their inferred divergence date of the most recent common ancestor was around 2000 (95% CI of the date: 1995 to 2006). The close genetic similarities of the Armenian isolates indicated that ST127 was potentially a dominant lineage in causing urinary tract infections in Armenia at the time of this study (January to September of 2019). In addition, we found that a French UPEC ESBL isolate was the only other isolate from the ENA archive that was part of the same genetic lineage and shared the most recent common ancestor with the Armenian isolates. Furthermore, we identified unique genes that were near known prophage genes in both the Armenian and French isolate clusters, which suggested that these genes were horizontally transferred via a prophage. These unique genes and the associated genomic regions were novel to the ST127 lineage but were present in other E. coli sequence types that were recovered from different human (including human urine samples) and animal sources. However, none of these isolates were shown to harbor all three genomic regions, suggesting a unique combination of genes that is only found in the Armenian and French UPEC ST127 isolate clusters. As there is a large Armenian community in France with relevant family links in Armenia, it seems plausible that the E. coli ST127 was transmitted between countries.

The most notable gene found in the Armenian and French ESBL E. coli ST127 lineage was the pore-forming toxin hemolysin E gene *hlyE* (also known as *clyA* and *sheA*). Hemolysin E has been identified across different species, including Salmonella typhi and Shigella flexneri, with a hemolytic action toward mammalian cells in anaerobic conditions (27). Moreover, the *hlyE* gene has been identified in APEC, UPEC isolates recovered from companion animals, and ExPEC isolates recovered from humans but has never been described as a virulence factor-associated cause of urinary tract infections from *in vivo* studies (4, 28–31). Moreover, we found that the haemolysin A gene *hlyA*, which is unrelated to *hlyE*, was ubiquitous in the Armenian E. coli ST127 isolates and has previously been described as an important virulence factor in causing pyelonephritis (27, 32, 33). Therefore, it is plausible that both genes can act synergistically to cause hemolysis in urinary tract infections; however, no such data have been reported up to date due to the rarity of isolates harboring both the *hlyA* and *hlyE* genes (29, 30). The *hlyE* gene region was determined to be a part of a prophage insertion region using the Prophage Hunter software package (34). We found that a small portion of the prophage region was relatively similar to an Stx2-converting phage Stx2a_F349, which is well-known for its horizontal transfer of the Shiga toxin (Stx) in Stx-producing E. coli (STEC). However, scientific evidence obtained under laboratory conditions has shown that Stx2-phages can also infect ExPEC isolates, although no evidence has ever been speculated of the prophage inserting the Shiga toxin gene into ExPEC isolates (35). Nevertheless, the BLAST search analysis of these genes in the *hlyE* prophage region identified two genes that were associated with prophages but did not originated from an Stx2-converting phage. These genes have shown homology to the Inovirus-type Gp2 family protein and the transcriptional regulator gene *alpA*, which is associated with the cryptic P4-like prophage CP4-57. Therefore, we can hypothesize that the prophage insertion region of *hlyE* originated from an unknown prophage species.

The further analysis of other unique gene regions found in the Armenian and French cluster isolates identified one of the unique genes as a prophage gene (considered to be unique if they exhibited less than <90% protein homology) and other unique genes as being adjacent to prophage genes in the bacterial chromosome. For example, we identified another gene that had homology to an Inovirus Gp2 family protein that was unique to our isolates as well as the IS3 family transposase IS2 near the TonB-dependent siderophore receptor gene *ireA*, which is involved in the increased uptake of iron within an iron-restricted environment, such as human urine (36). The *ireA* was found in five other isolates that were retrieved from ENA and were recovered from companion animals and human urine samples, but these isolates showed no direct phylogenetic or geographical linkage to each other. Moreover, we also found that other *ireA* ST127 isolates had an Inovirus Gp2 family protein gene; however, these genes only shared 87.24% homology at the protein level. The difference in homology may be linked to the high mutation rates within filamentous phages due to them having single-stranded DNA genomes (37). We also found a difference between the genome regions of the Armenian and French isolates cluster, as they had insertion sequence IS3 family transposase IS2 elements between the *ireA* and Inovirus Gp2 family protein instead of a hypothetical protein, as found within the other ST127 *ireA* isolates. Moreover, a truncated version of the IS255 family transposase ISod4 was not truncated in the other ST127 *ireA* isolates. The insertion sequence acquisition and changes within the Armenian and French isolates cluster may have a role in the immobilization and stability of the prophage region, which may suggest why we did not find other E. coli ST127 clusters containing the *ireA* gene (38, 39).

Furthermore, the Armenian and French isolates cluster had a genomic region that contained 11 unique genes that were adjacent to the prophage integrase gene *intA* and the tRNA-phe gene. Downstream of these genes were the unique genes to the Armenian and French cluster, including a peptide ABC transporter substrate-binding protein, the lactate utilization protein operon *lutABC* (also known as *ykgEFG* in E. coli), and the endonuclease restriction modification operon *hsdMSR*. To our knowledge, there have been no reports of the involvement of these genes in UPEC growth and survival in the urinary tract. Nonetheless, we can hypothesize that the peptide ABC transporter substrate-binding protein gene, *lutABC* operon, and *lutP* permease, may have a role in utilizing lactate as an energy source, along with other metabolites found in the urine; however, further studies are required (40–42). In addition, the *hsdMSR* operon may have initially acted as a superinfection exclusion mechanism to prevent secondary bacteriophage infections (43). Moreover, the *hsdMSR* operon may act as a gene regulation mechanism within the urinary tract via DNA methylation (44, 45).

Although we were not able to find a complete arsenal of prophage gene integration within all of these genomic regions, we hypothesize that the most likely mode of horizontal transfer for these genomic regions was through a prophage and that the prophage genes have decayed over time due to point mutations and deletions to remove unwanted and toxic genomic features, leaving behind remnants of prophage genes (46). Furthermore, based on the date of divergence (1966 date CI: 1956 to 1977) of the United Kingdom isolates recovered from blood cultures in 2011, which were the closest related isolates to the Armenian and French cluster, we cannot determine when these genomic regions integrated into the Armenian and French E. coli ST127 lineage or whether it happened before or after they diverged from the United Kingdom isolates.

Although we did not find antibiotic resistance gene mutations and plasmid replicons that were ubiquitous and unique to the Armenian isolates, we did identify that the quinolone resistance protein gene *qnrD1* was unique to ARM88 and that the plasmid replicon genes and IncI2_1 were unique to ARM66. The *qnrD1* is generally rare in E. coli isolates and is more commonly identified in Proteus and Providencia species, whereas the IncX3_1 and IncI2_1 are generally associated with the spread of carbapenem and colistin antibiotic resistance genes (47–52). However, we could not link them to any plasmids because we could not identify a known plasmid replicon with an ARM88 plasmid sequence. In addition, we did not detect any antibiotic resistance gene that was associated with carbapenem and colistin antibiotic resistance in either ARM64 or ARM66. In correlation with the phenotypic data, we determined that ARM88 *qnrD1* showed known resistance to fluoroquinolone antibiotics (noroflaxin and levofloxacin). Intriguingly, we found 46 antibiotic resistance determinants that were ubiquitous in all of the E. coli ST127 isolates that were studied, which coincides with an analysis by Goldstone and Smith (2017) that described 50 antibiotic resistance genes, which may play a role in providing basal-level resistance toward a diverse number of antimicrobial compounds, as being part of the core resistome (≥95% of the isolates) (53). However, Goldstone and Smith (53) also pointed out that some of these core antibiotic resistance genes may not be involved in antibiotic resistance in E. coli due to a lack of evidence in the literature, but they have been involved in antibiotic resistance in other bacterial species. Our findings indicate that the β-lactam and cephalosporin antibiotic resistance phenotype in the Armenian isolates can be attributed to the ESBL associated genes *bla*_CTX-M-3_, *bla*_CTX-M-236_, and *bla*_TEM-1_. However, we were unable to determine the antibiotic resistance genotype that confers phenotypic resistance to the aminoglycoside antibiotic amikacin in ARM88 resistance to the fluoroquinolone antibiotics norfloxacin and levofloxacin in ARM75, or intermediate resistance to the carbapenem antibiotics as well as imipenem resistance in ARM66. Moreover, the antibiotic resistance phenotypes observed in our study were consistent with the findings of previous studies that report that ST127 are sensitive toward a wide range of antibiotics, compared to other UPEC sequence types, and that the aminoglycoside and fluoroquinolone antibiotic resistances as well as ESBL production were not commonly found in ST127 isolates (5, 54, 55).

The main limitation of this study is its small sample size; however, this is the first genomic analysis of ESBL UPEC ST127 isolates recovered from patients in Armenia. These isolates were recovered from different hospitals in different regions of Armenia but belonged to the same genetic lineage and share a recent common ancestor. In addition, all of the isolates shared virulence and metabolic genes that were acquired via horizontal transfer and were not found in other E. coli ST127 isolates, except in one phylogenetically closely related UPEC isolate from France. Further whole-genome sequencing surveillance is necessary to better understand the molecular epidemiology of the Armenian isolates and the roles of some of these genes as well as to determine whether ST127 is the dominate lineage causing urinary tract infections in Armenia. Such surveillance studies will contribute to global efforts to tackle antibiotic resistance, as bacteria carrying AMR genes do not recognize borders.

## MATERIALS AND METHODS

### Bacterial isolation and identification.

12 E. coli isolates were received from the medical microbiology laboratories of 5 hospitals in Armenia between January and August of 2019. All isolates were recovered from urine specimens of hospitalized patients. The isolates were identified as E. coli using matrix-assisted laser desorption ionization-time of flight mass spectroscopy (MALDI-TOF-MS), as described previously (56). 4 out of 12 isolates that were identified as E. coli ST127 were selected for the purposes of this study.

### Antibiotic susceptibility testing.

All 12 isolates were tested for their antibiotic susceptibility to a panel of 11 antibiotics, including ampicillin (10 mg), piperacillin-tazobactam (30/6 mg), amoxicillin and clavulanic acid (20/10 mg), ceftazidime (10 mg), cefepime (30 mg), norfloxacin (10 mg), levofloxacin (5 mg), amikacin (30 mg), imipenem (10 mg), meropenem (10 mg), and chloramphenicol (30 mg) (Mast Group, Merseyside, UK), using a disk diffusion method, according to the European Committee on Antimicrobial Susceptibility Testing protocol (57). The antibiotics chosen were those that are the most frequently used in clinical settings in Armenia. E. coli isolates were identified as “ESBL-producing” upon the confirmation of their resistance to cefepime and ceftazidime antibiotics.

### Genome sequencing and assembly.

All of the E. coli isolates were whole-genome sequenced using an Illumina HiSeq platform. However, for the purposes of this study, WGS analyses were conducted for only 4 isolates belonging to ST127. Genomic DNA was extracted using a TIANamp Bacteria DNA Kit (Tiangen, China) and the paired-end sequencing libraries were constructed using Nextera XT DNA Sample Preparation Kits or a TruSeq DNA HT Sample Prep Kit (Illumina, USA), following the manufacturers’ instructions.

The quality of the short-reads was analyzed using fastQC, and low quality reads were trimmed using the Trimmomatic software package (58). The trimmed reads were *de novo* assembled using SPAdes (26).

### Genome selection for phylogenetic and genomic comparison.

To conduct a comparative genomic analysis of the E. coli ST127 isolates recovered in this study, 168 draft E. coli ST127 genomes (Table S1) were obtained from the ENA database (accessed January 2022). The selection criteria of the isolates included the date and source of isolation and the country of origin. To select only the ST127 genomes among the 12 E. coli isolates recovered in this study and those retrieved from the ENA database, we screened the draft E. coli genomes using mlst (https://github.com/tseemann/mlst, accessed July 2021) with the Achtman typing scheme from pubMLST (accessed January 2022).

### Phylogenetic analysis.

To construct a core SNP maximum-likelihood (ML) phylogenetic tree of the E. coli ST127 isolates, we first aligned all of the isolates against the reference E. coli ST127 genome EC536 (accession no. NC_008253.1) using the Parsnps alignment software from the Harvest suite (59). Then, recombination was removed from the aligned sequences using Gubbins, and a phylogenetic tree was constructed from the recombination-free alignment using IQtree v2.1.2 (60). To select the best model for the construction of the phylogenetic tree, we used ModelFinder and set the ultrafast bootstrap replication to 1,000 (61, 62). Finally, the phylogenetic tree was visualized using iTOL (63). The SNP-distances between the isolates core genomes were worked out from the recombination-free alignment using the SNP-dist software package (https://github.com/tseemann/snp-dists).

A Bayesian dated maximum clade credibility (MCC) tree was reconstructed using BEAST v1.10.4 (64). A GTR empirical substitution model, as determined by ModelFinder, was used for the Bayesian analysis and was set with a Bayesian skyline strict clock model with the MCC chain set to 100 million. 2 independent runs trees were combined using LogCombiner with a 10% burn-in and an MCC tree was constructed from the combined trees using TreeAnnotator. The MCC tree was visualized using the FigTree software package (http://tree.bio.ed.ac.uk/software/figtree/).

### Genome annotation.

The genomes of the E. coli ST127 isolates were annotated using Prokka (65). Virulent gene markers and plasmid replicons were screened using the Abricate software package (https://github.com/tseemann/abricate) in conjunction with the VirulenceFinder and Virulence Factor Database combined for virulence detection and the PlasmidFinder database for the identification of plasmid replicons (accessed January 2022) (66–68). Antibiotic resistance genes/mutations were screened using the Resistance Gene Identifier software in conjugation with the Comprehensive Resistant Antibiotic Resistance Database (accessed January 2022) (69). The isolates were serotyped by ECtyper (70).

### Pangenome and unique gene analysis.

The pangenome combining both the Armenian and ENA E. coli ST127 isolates was constructed using Roary with the BLASTP percentage identify cutoff set to 90% (71). Unique genes within the different E. coli ST127 groups were identified using Scoary (72). Unique genes and genes within their loci were further annotated via a BLAST against the UniProt database and the NCBI nucleotide collection. MAFFT was used to identify individual protein percentage similarities (73). The genomic region comparison was visualized using Mauve v 2.4.0 (74).

The MOB-suite was used to reconstruct plasmid sequences from draft genomes to determine whether unique genes were plasmid-borne (75). Integrative and conjugative element genomic regions were detected using ICEfinder with the ICEberg 2.0 database (76). Prophage Hunter was used to identify the prophage regions in the draft genomes to determine whether unique genes were within the prophage insertion region (34). The horizontal gene transfer regions that contained these unique genes were compared to other E. coli isolates via a sequence similarity search using NCBI blast against the ENA sequence archive (77).

### R programs.

The grouping of isolates into subclusters within the SNP core whole-genome alignment was conducted using the hierarchical clustering R package “RhierBAPS” (78). In addition, a hierarchy cluster heat map of antibiotic resistant genes/mutations, accessory genes, and plasmid replicons was constructed using the pheatmap R package (https://cran.r-project.org/web/packages/pheatmap/index.html).

### Data availability.

The short-read data were deposited in the ENA under the study PRJEB51925. Individual sequence file accession numbers are included in Table S2.
